# Experiences and satisfaction with intensive community care services for children and adolescents with severe mental health disorders: a mixed-methods systematic review

**DOI:** 10.1007/s00787-026-03147-8

**Published:** 2026-08-13

**Authors:** Ben Hoi-Ching Wong, Jasmine Burrage, Mona Albazi, Priya Hornby, Opeyemi Atanda, Dennis Ougrin

**Affiliations:** 1 East London NHS Foundation Trust, London, UK; 2 Queen Mary University of London, London, UK; 3 University of Liverpool, Liverpool, UK; 4 London South Bank University, London, UK; 5 Institute of Mental Health, Ukrainian Catholic University, Lviv, Ukraine

**Keywords:** Community mental health, Service design, Experience and satisfaction, Children and young people, Alternatives to inpatient, Parental experiences

## Abstract

Intensive community care services (ICCS) are community-based alternatives to inpatient care for young people with severe mental health disorders. This systematic review investigated the experiences and satisfaction associated with ICCS. Nine electronic databases were searched from inception to August 2025 for studies on children and adolescents under 18 years receiving mental health care from ICCS. Primary outcomes were experiences and satisfaction from the young people, parents, or clinicians. Quantitative satisfaction data were qualitised before integrating with qualitative data. The synthesis used a meta-aggregative approach to produce statements to inform practice. Nine studies from eight articles were included: two qualitative, three mixed-methods, and four quantitative studies. No studies examining clinician experiences were identified. Positive experiences were generated when ICCS: (a) provide structured emotional and practical support to caregivers; (b) facilitate strong therapeutic relationships across the young person-family-clinician system through frequent, flexible, and community-based contact; (c) offer multidisciplinary expertise and specialist interventions; (d) support sustained improvements beyond treatment by embedding therapeutic work in real-world contexts; (e) actively manage extra-therapeutic factors and family involvement to avoid undermining young people’s autonomy or sense of agency. Parents were particularly satisfied with ICCS due to accessibility, responsiveness, intensity of contact, and reduced hospitalisation. ICCS models successfully generate positive experiences in service users. Satisfaction was generally higher than routine care and particularly pronounced amongst parents. Future studies should explore staff perspectives and specific elements of satisfaction. These findings have implications for service design, workforce training, and balancing family involvement with young people’s autonomy.

**Supplementary Information:**

The online version contains supplementary material available at https://doi.org/10.1007/s00787-026-03147-8.

Mental health conditions are a major cause of morbidity for children and young people globally. In England, one in five children had at least one diagnosable mental health problem [1]. There has been a significant increase in acute presentations of mental health disorders in recent years [2]. Limitations of psychiatric hospitalisation [3–5] call for alternative service models that provide equivalent levels of support in the community.

One growing research focus is the development of intensive community care services (ICCS) [6]. ICCS encompass a variety of community-based mental health services that are used as an intermediate step between general outpatient therapies and inpatient admission, providing high-intensity, frequently accessible care in the community for people who have severe mental health difficulties that pose risks to themselves or others [6].Well-known ICCS models, such as assertive community treatment (ACT) [7], supported discharge service (SDS) [8], multisystemic therapy (MST) [9], have all shown promising clinical effectiveness, such as reducing repetition of self-harm and externalising psychiatric symptoms. Although the theoretical mechanisms underpinning ICCS have not been fully studied, their emphasis on intensive, relational work in real-world contexts is consistent with humanistic and ecological systems perspectives on youth mental health care [10]. These in turn may alleviate social isolation in young people and minimise disruptions to education [6]. Systematic reviews of ICCS models [11, 12] in children and adolescents found that they have at least comparable effects on patient functioning compared to inpatient hospitalisation, but at a lower economic cost, demonstrating their cost-effectiveness and practical potential.

To our knowledge, no systematic review has examined the qualitative experiences or satisfaction with ICCS. A review investigating the qualitative experiences of alternative care to inpatient settings found that children and adolescents were generally more satisfied with alternative care, whereas parents experienced emotional burden when engaging with services [13]. However, the studies included in the review were any non-inpatient alternatives, including urgent, and often brief interventions such as emergency room crisis provision and mobile applications. It remains unclear how service users experience the more intensive, longer-term treatment models developed to fully substitute traditional mental health services (including inpatient and routine community care). Understanding these experiences is important because satisfaction is not only an indicator of service quality, but it also predicts treatment adherence [14]. This is particularly relevant in youth mental health, where both young people’s and family members’ experiences affect their engagement with services. It is also important to understand the clinicians’ qualitative experiences, as ultimately, healthcare research aims to offer practical guidance that clinicians can incorporate in their practice.

This review aimed to address this important gap in the literature. Our primary objective was to explore what constituted positive experiences of children and adolescents receiving ICCS support as well as their carers, in order to inform improvements in service delivery. Our secondary objectives were to investigate the satisfaction level of ICCS to understand the clinicians’ perspectives on providing service in ICCS.

## Methods

The protocol of this systematic review is registered and available on PROSPERO (CRD42024541495). We adopted a mixed-methods review, convergent integrated approach, in accordance with the Joanna Briggs Institute (JBI) methodology [15], to incorporate both qualitative and quantitative components into qualitative findings of experiences and satisfaction. A meta-aggregation framework was used to produce synthesised statements that describe the experiences with ICCS, providing insights for future service development. This review was reported according to the Preferred Reporting Items for Systematic reviews and Meta-Analyses (PRISMA) guidelines (see Appendix S1 for the PRISMA checklist).

## Eligibility criteria

We included all studies on children (under 18 years) who require intensive outpatient mental health support and received intervention from any ICCS model (defined below) for their mental health conditions. Studies where adults accounted for more than 50% of the patients receiving the service were excluded. Meta-analyses, systematic reviews, books, and grey literature are excluded study types, though individual studies identified through them remained eligible for inclusion. Although any treatment service as usual (e.g. inpatient admission and generic CAMHS services) is usually compared or controlled in studies on ICCS, this was not a criterion of eligibility in this review.

## Outcomes

The primary outcome of interest was qualitative experiences or satisfaction among service users (young people and/or their parents) and clinicians regarding the ICCS service. Experience refers to participants’ qualitative reports of processes and interactions within ICCS. This includes but is not limited to: accessibility, therapeutic engagement, and perceived impact on daily functioning. For example, details of positive experiences could be captured in interviews, focus groups, or detailed questionnaires when a service user explains the aspects of care they find helpful or beneficial to them or their family. Satisfaction refers to overall evaluation of service quality, typically assessed using rating scales, or in binary questions.

### Intensive community care services (ICCS)

Our search focused on mental health service models classified as ICCS. ICCS teams typically provide community-based mental health support to young people, as an alternative to inpatient admission or regular community services [6, 11] Although not compulsory or exhaustive, ICCS often include the following key features [6]: (1) support primarily delivered in the community settings; (2) at least some out-of-hours support is provided; (3) a minimum of 90% of patients receive direct clinical contact at least twice per week; (4) minimum 4 full-time equivalent (FTE) staff members, with at least one professional; (5) maximum caseload should be approximately 5 clients per FTE clinician; (6) actively support and promote engagement of the patient’s wider family and informal support network; (7) flexible and persistent approach to engaging patients; and (8) regular monitoring and evaluation of the progress of each patient.

## Searches and study selection

We searched for peer-reviewed publications in the English language on the following electronic databases: MEDLINE, Embase, Global Health, PsycINFO, PubMed, CINAHL, CT.gov, and ICTRP. The searches were performed using OVID interface and the Cochrane Library, without any restriction on publication date.

Based on an initial scoping search and previous review and professional consensus research by our team [6, 11], we developed a keyword mapping search strategy, covering a combination of following key terms and their synonyms/MeSH descriptors, e.g. child*, adolescen*, mental health, psychiatr*, intensive community, assertive, home-based, outpatient, crisis care, experience, satisfaction, qualitative. Full strategy is presented in Appendix S2. The limit was set to include only English literature. The electronic search was conducted on 11th December 2024, with a further final search performed on 1 August 2025 to capture any updated publications.

All identified studies were imported into EndNote 20 software, and duplicates were removed. Selection and screening were performed by four co-authors (BHCW, JB, MA, OA). Each identified study was screened independently by two members of the reviewer team. Any disagreement was resolved by consensus, and where this was not possible, by a third team member. Reasons for exclusion were recorded.

## Data extraction and synthesis

The present review focused on the experiences and satisfaction. Two authors (JB and MA) extracted the data independently, checked by the lead author. Data extraction followed a priori template (Appendix S3) developed to capture the study and service characteristics, as well as the main qualitative findings. Study teams of the included studies were contacted to obtain any important information that was missing. Where experience or satisfaction was measured quantitatively, the extracted data were qualitised according to JBI guidance and converted into textual descriptions to facilitate integration into the synthesis [15].

Our data synthesis adopted the meta-aggregation approach [16]. This method was chosen in order to systematically identify patterns of evidence across all studies, whilst preserving the original meanings established by the original authors and limiting influence from the reviewers. Each qualitised statement was treated as an extracted finding at the synthesis stage before grouping into categories. Findings from each included study were first extracted individually by each reviewer, using a structured data extraction template, then organised manually into preliminary categories based on similarity of meaning. The reviewers (BHCW, JB, MA, PH) discussed the extracted findings and their references in the respective studies and identified any emerging patterns, i.e. recurring or contrasting findings across multiple studies related to any aspects of ICCS delivery and resulted experiences to the stakeholders. A hierarchy of findings was generated in the process by first extracting findings from included studies with minimal paraphrases. The level of plausibility for each finding was assessed according to JBI guidance, and any unsupported findings were removed. Findings were rated as unequivocal when there was no alternative interpretation to the data which provided logical support for the resulted conclusion, and equivocal when the findings were supported but potentially open to more than one interpretation. Unequivocal and equivocal findings were given equal weights in synthesis, but indicate whether they were open to alternative interpretation in the original studies. The findings were then categorised based on similarities in meanings. Synthesised findings were finally developed into statements to produce meaningful insights that inform clinical practice.

## Quality assessment

Critical appraisal of study quality was conducted by adhering to the JBI guidance of mixed-method systematic review [17], using JBI checklists of corresponding study types [16, 18, 19]. For quantitative studies, only satisfaction outcomes were assessed. All studies, regardless of methodological quality, underwent data extraction and synthesis. Quality appraisal was conducted focusing on the aspects of methodological rigour most relevant to the objectives of the current review. For quantitative studies, this included the design, measurement, and reporting of satisfaction outcomes; for qualitative and mixed‑methods studies, this included clarity of data collection and analysis procedures for experience and satisfaction data, and the coherence between data, interpretation, and conclusions.

## Results

### Study selection

**Fig. 1:**
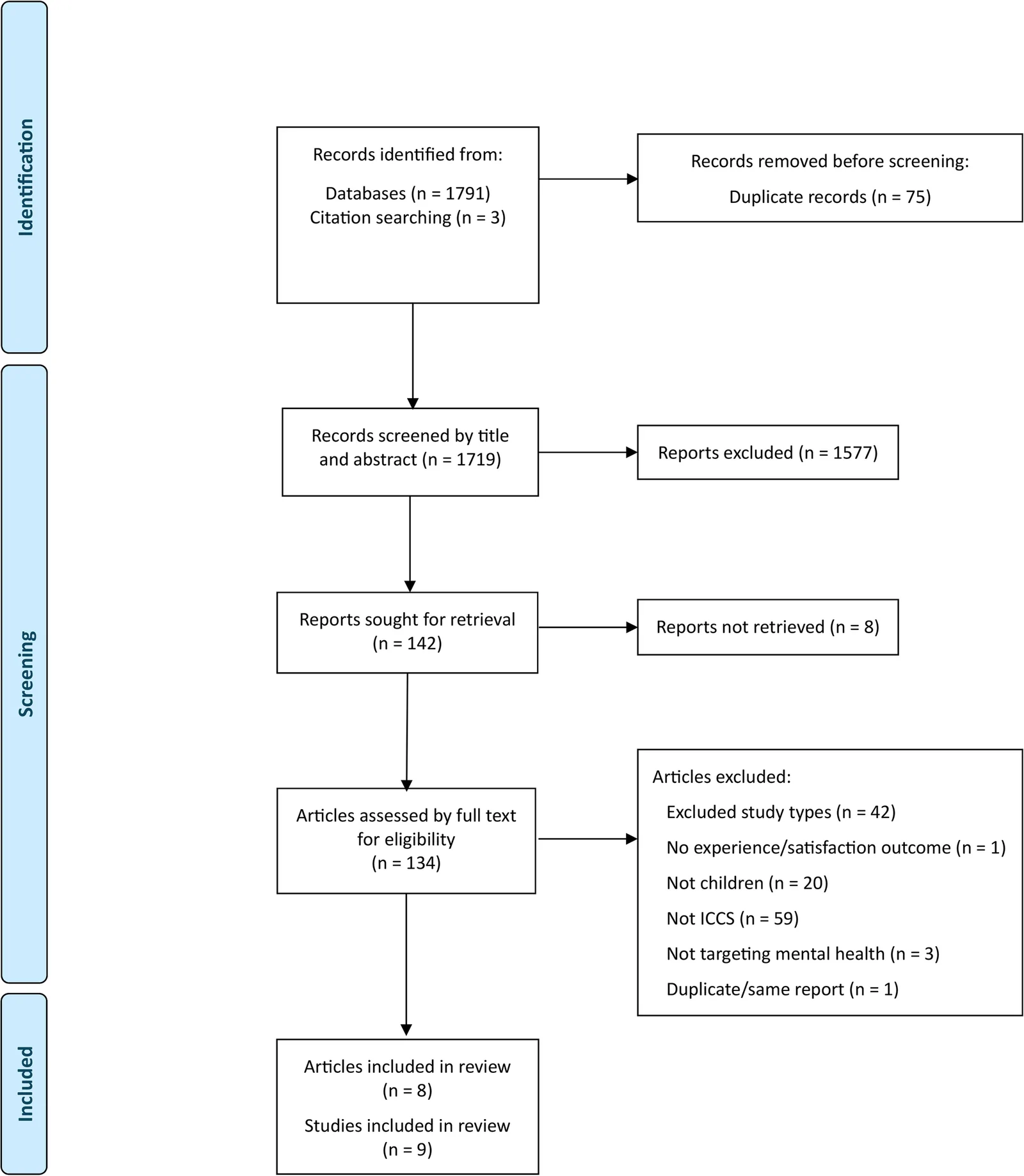
PRISMA diagram

Our search returned 1794 results. After deduplication, abstract and title screening against eligibility criteria was conducted for 1719 publications, 142 of which proceeded to full-text screening. The most common reasons for exclusion were ‘not ICCS’ (n = 59), followed by ‘excluded study type’ (n = 42). Other exclusion reasons include ‘not children’ (n = 20), ‘not targeting mental health’ (n = 3), ‘no experience/satisfaction outcome’ (n = 1). Of nine eligible articles, two were identical reports of the same study. In total, nine studies from eight articles were included in the evidence synthesis. See Fig. 1 for the PRISMA flow diagram.

### Study characteristics

The characteristics of the nine included studies are presented in Table 1. Four of the studies were conducted in the UK, two in Germany, two in the United States, one in Australia and one in Canada. All the studies were published between 1998 and 2021, with 6 out of 9 being published over 2009. Two studies [20, 25] were qualitative studies conducted using semi-structured interviews, three were nested qualitative studies in mixed methods research [22, 26] using both questionnaires and semi-structured interviews, and four were quantitative [9, 21, 23, 24] including satisfaction questionnaires. Five studies included adolescent participants, and five included parents. No studies examining clinician experience in ICCS were identified.

**Table 1:** Characteristics of included studies

Author, Year	Country	Intervention model	Key features	Study type; Data collection methods	Qualitative analysis approach	Sample size & Characteristics (*N*)
Conti et al., (20)	Australia and New Zealand	Family-Based Therapy (FBT)	Three-phase treatment over about 12 months/20 sessions: Weight restoration with parent engagement, empowering adolescents, re-establishing autonomy, developing a healthy identity, and address psychological issues. Conjoint family sessions or separated family therapy.	Qualitative; Semi-structured interview	Inductive thematic analysis	14 patients (14–27 years old) with anorexia nervosa who previously received FBT
Measelle et al., (21)	United States	Family Mosaic system (case management)	Interagency case management, providing community-based, individualised, family-centred service and coordination of care for severe emotional disturbance children. Average caseload of 10–12 families per case manager. Average 5–6 patient contacts per month. Crisis support and long-term service linkage available.	Quantitative; Questionnaire (Family Satisfaction Survey)	N/A	51 caregivers of patients with severe emotional disturbance
Fonagy et al., (22)	United Kingdom	Multisystemic Therapy (MST)	Comprehensive, individualized treatment in community addressing youth, family, peers, school. Intensive engagement focusing on improve parenting skills and the parent–child relationship, improve communication problems, foster support from social networks, encourage the young person’s participation in education and reduce any associations with delinquent peers. Average 5–6 months treatment duration. Therapist caseload of 4–6 families. Weekly supervision and quality assurance.	Mixed methods; Semi-structured interviews, satisfaction measure in questionnaire (FACES-IV) was also extracted	Study 1: Framework analysis Study 2: Interpretative phenomenological analysis	Study 1: 14 young people and 13 caregivers Study 2: 32 young people
Henggeler et al., (9)	United States	Home-based MST	Modified MST for psychiatric emergencies, primarily home-based. Reduced caseload of 3 families per clinician. 24/7 crisis coverage with crisis caseworkers and psychiatry residents. Daily supervision initially, reduced to 3 times per week. Comprehensive crisis planning and assessment of systemic risks and strengths, and collaborating with family members to design interventions for change. Expanded team included child and adolescent psychiatry residents and crisis caseworkers providing 24/7 coverage, and brief hospitalisation (mean 3.8 days) possible during treatment.	Quantitative; Questionnaire (Family Satisfaction Survey)	N/A	113 adolescents (10–17 years old) with severe mental illness symptoms requiring hospitalisation
Preyde et al., (23)	Canada	(Intensive) home-based alternative	Intensive, home-based support, services comparable to residential care intensity. Services include family support services, provided for 3–6 months and include a strength-based approach to family counselling, crisis intervention, family and individual counselling, after-hours crisis support, specialty services, and outreach and parent support/education services.	Quantitative; Satisfaction ratings in interviews	N/A	88 caregivers
Herpertz-Dahlman et al., (24)	Germany	Home treatment (HoT)	Shortened inpatient psychological stabilisation (4–8 weeks) followed by 4 months home treatment. Multi-disciplinary team with up to 4 home visits per week. Provides individual and group psychotherapy, focusing on social rehabilitation in conjunction with weight restoration.	Quantitative; Questionnaire (ZUF-8)	N/A	32 patients (12–18 years) with anorexia nervosa. 42 individuals (21 patients and 21 carers) completed the survey
Boege et al., (25)	Germany	Home treatment (HT)	Stepped Care; shortened inpatient stay enabled by 12–16 weeks of intensive home treatment integrating therapeutic, clinical, and case management services with up to three weekly sessions. Crisis management available 5 days/10 hours per week by the team, plus 24/7 doctor on call.	Qualitative; Semi-structured interview	Inductive thematic analysis	51 caregivers
Gowers et al., (26)	United Kingdom	Specialist outpatient programme	Specialist outpatient programme consisting of individual cognitive behaviour therapy, dietary advice, parental counselling and multimodal feedback of weight and self-report measures. Treatment duration of 6 months.	Mixed methods; Focus group and satisfaction questionnaires	Inductive thematic analysis	215 participants (12–18 years old) with anorexia nervosa in total. Qualitative focus groups included 21 youths

### Quality of included studies

All qualitative studies [20, 22, 25, 26] demonstrated moderate dependability, with none fully meeting the criteria regarding the researchers’ cultural or theoretical background (Q6) and the influence of the researcher on the research and vice versa (Q7). Credibility was unequivocal for all qualitative studies, meaning the findings were definitive and accompanied by an illustration that was beyond a reasonable doubt. The quantitative studies generally demonstrated low risk of bias. The trial by Henggeler et al. [9] did not have participant and clinician blinding to treatment allocation. However, blinding is not possible in research on ICCS models, as different groups are, by nature, treated in different settings. Studies by Herpertz-Dahlmann et al. [24] and Preyde et al. [23] met 7/9 and 8/9 applicable criteria, respectively, lacking multiple pre- and post-intervention outcome measurements. The study by Measelle, Weinstein, and Martinez [21] met 3/5 applicable criteria, missing a control group and multiple measurements.

We also assessed the individual credibility of each original finding based on the congruency of the original interpretations and their supporting data. These ratings are included in the synthesis table (Table 2). Synthesised findings composed of only unequivocal findings represent high confidence in the output produced, while a mixture of unequivocal and equivocal findings indicates moderate confidence [27] No finding was unsupported by original data and removed from synthesis.

**Table 2:** Summary table of synthesis

Finding	Category	Synthesised finding
• Parents valued family work and being involved in treatment (U) • Having professional support/help in everyday life at home was the most helpful element (U) • Nearly all parents in the intervention group perceived a gain in own competency (U)	Supports for caregivers	Emotional and practical support for caregivers enhances satisfaction and competency
• Importance of individual therapeutic relationship (U) • Most parents in the intervention group described positive therapeutic relationship between child and therapist (U) • Positive relationships with therapists and learning techniques for conflict management and communication contributed to initial change (U) • Importance of positive interpersonal relationships, reduced detriment of mixing with other patients (U)	Positive interpersonal relationships	ICCS provide good foundation of positive relational dynamics between young person, family and clinician
• Therapists advocating for parents reduced their sense of being alone (E) • The child felt more connected to their parents (U) • Family relationships and effective family communication marked positive trajectories of change (U)	Improved parent-child relationships	
• Importance of expertise of clinicians in specialist outpatient (U) • Patients were able to trust the expertise of the clinicians (E)	Importance of quality of clinician expertise	The range and level of clinician expertise are fundamental to the ICCS models
• Importance of availability of dietetic therapy in specialist outpatient (U) • Availability of specific therapy (U)	Having access to specialist therapy or intervention	
• Improvement after discontinuing treatment was reported more in the control group than in the intervention group (E) • Provided positive work experiences that contributed personal growth (U) • Continued use of techniques and generalisability contributed to sustained changes (U) • Helped develop meaningful peer relationships (U) • Prepared for child-adult transition (U)	Continuity and generalisability of improvements beyond treatment	ICCS meet patient needs and generate sustain improvements beyond treatment
• More patients in the intervention group needed no further treatment than in the control group (E)	Patients’ needs are sufficiently addressed	
• Extra-therapeutic factors (e.g. individual or environmental factors) could promote or hinder positive change (U)	Non-therapeutic factors influence therapeutic outcomes	Effective treatment should consider how to navigate non-therapeutic factors and mitigate potential drawbacks of involving families
• Family distress and conflict was experienced (U) • Difficulty for child to negotiate personal agency and voice in treatment, especially where parents were involved (U) • Young people’s individual psychological difficulties and distress were overlooked (U)	Unintended conflicts and unmet needs when involving families	
• Higher satisfaction in both adolescents and parents with the specialist outpatient treatment than standard outpatient (U) • Treatment satisfaction was more pronounced in caregivers (U) • Greater monthly contact and lower rates of child psychiatric hospitalisation were two independent factors of parent satisfaction (U)	Treatment satisfaction	ICCS result in higher satisfaction compared to alternative care, especially amongst parents

Note: Plausibility of results recorded for each finding (U: unequivocal; E: equivocal)

#### Synthesis findings

Qualitative findings, including qualitised quantitative findings, were extracted and synthesised from all included studies (Table 2). These findings formed 9 categories in total, out of which six synthesised statements were produced to provide implications for clinical practice.

#### Synthesised finding 1: Emotional and practical support for caregivers enhances satisfaction and competency

In general, the support and advice generated by ICCS were found to be beneficial to caregivers of the children and adolescents receiving the service. Therapies that were family-oriented were perceived as helpful, as parents valued being kept informed and involved throughout treatment [26]. ICCS tend to provide structured emotional and practical support to caregivers, including elements of psychoeducation, skills coaching, and shared problem-solving. Having professionals directly helping to tackle daily challenges not only added to the practical assistance but also provided important emotional reinforcement for caregivers. A sense of ‘fighting the battle together’ was commonly reported. ICCS put systems that empower caregivers to manage the difficult mental health conditions of their children, and caregivers generally felt more confident in their own independent ability to continue to apply the skills they learned [25].

#### Synthesised finding 2: ICCS facilitate strong therapeutic relationships across the young person-family-clinician system through frequent, flexible, and community-based contact

Positive interpersonal connections underpin the work of many ICCS models and are a frequently reported benefit. The importance of maintaining a positive therapeutic relationship was identified, with benefits found for both the clinician and patients. The intensity and individualised nature of ICCS allowed patients feel more listened to and understood. Meanwhile, it was easier for clinicians to deliver treatment plans that were adhered to. Patients could leverage the positive relationship with clinicians into further improvements of interpersonal relationships. For example, learning conflict management and communication skills has proved to be useful for patients in contributing to initial change [22]. Children and adolescents also found lower negative impacts of being with peers with serious mental disorders, as ICCS often resulted in reduced time or completely bypassing treatment in stressful inpatient settings [26].

The positive relationships extend to between the child and their family and caregivers, commonly seeing an increase in connectivity within the parent-child relationship [20]. Children reported a sense of ‘No one was ever going to give up on me’ when they directly witnessed the active engagement of parents in the treatment. Key areas of improvement from ICCS included effective communication within the families, highlighting the result of a positive relational dynamic between caregiver and child from the treatment process [22].

#### Synthesised finding 3: The availability of multidisciplinary expertise and specialist interventions within ICCS is a core determinant of positive experiences

The clinicians’ expertise in ICCS models is central to their quality and the success of treatment in order to meet the specific needs of patients. Children and adolescents repeatedly reported the importance of knowing they are being treated by experienced professionals as well as being able to trust them. This was the result of having clinicians who could not only tangibly demonstrate specific ability and knowledge, but also sympathise with the patients, making them inclined to engage in treatment [26].

Access to specialist therapy in an outpatient setting was also identified as fundamental to ensuring the efficacy of ICCS. ICCS are predominantly multidisciplinary, commonly including psychiatrists, psychologists, mental health nurses and social workers, with each trained in various therapeutic modalities [6]. Therefore, the teams often comprise different skillsets, enabling a variety of interventions that can match the needs of individual patients, such as dialectical behaviour therapy, cognitive behavioural therapy, family therapy, solution-focused brief therapy, etc., supplemented by psychotropic medication prescription if needed. For example, a specialist outpatient treatment for anorexia nervosa might be able to provide dietetic therapy at home, circumventing the need for children and adolescents who are severely ill to be admitted [26].

#### Synthesised finding 4: ICCS meet patient needs and generate sustain improvements beyond treatment

ICCS support sustained improvements beyond the treatment period by embedding therapeutic work in real-world contexts, promoting skill generalisation, and preparing young people and caregivers to manage future challenges independently. There was often a focus on continuity, with some ICCS models prepared adolescents for transitioning into adulthood by equipping them with essential strategies for managing their mental health in daily lives, as well as practical experience, such as work experiences [22].Developing skills such as social skills in the community could also help develop meaningful peer relationships in real-life settings, which could in turn facilitate a positive cycle, creating a sustainable support system. Overall, service users felt that the improvement could generalise beyond treatment as individuals can take these skills and techniques away and help further develop meaningful peer relationships. This was reinforced by findings such as lower rehospitalisation or more patients in the ICCS group needed no further treatment than in the control group [25], indicating that patients’ needs had been sufficiently addressed.

#### Synthesised finding 5: Effective treatment should consider how to navigate non-therapeutic factors and mitigate potential drawbacks of involving families

There are factors beyond treatment were identified to possibly hinder or promote positive changes. These were described as a ‘double-edged sword’ that can in turn influences the therapeutic outcomes and experiences of ICCS. Factors on an environmental level, such as changing schools, involvement of antisocial peers, or community safety, as well as individual factors, such as personal maturity and parental mental health, could all contribute to potential positive or negative impacts [22].

Although involving family in therapy has been found to be helpful in improving parent-child relationships, unintended conflicts were reported in some cases. Increasing the responsibilities of parents in treatments sometimes resulted in situations where children struggle to negotiate personal agency within their treatment [20], exacerbating any existing conflicts and distress they experience. A young person described this experience as feeling like ‘a monkey in cage’, as they were reduced to their mental illness whilst parents were doing everything. There were also reports of feeling unable to verbalise the underlying distress because of existing discord with parents, who incidentally took on a bigger role in the treatment. These highlight the importance of maintaining the perspective that, despite all the associated benefits of family engagement, caregivers should only be involved as a supportive system, and not the primary service users.

#### Synthesised finding 6: ICCS result in higher satisfaction compared to alternative care, especially amongst parents

Service users reported higher satisfaction with the ICCS support they received. This was backed by both qualitative and quantitative findings. Compared to alternative care such as standard community care or inpatient, the accessibility and responsiveness of service, greater monthly clinical contacts in ICCS, and lower rates of child psychiatric hospitalisation contributed to the higher service satisfaction [21]. Service users also highlighted the respectfulness and helpfulness of staff, and the quality of services they received [23]. Although favourable opinions were registered in both children and parents, the latter tended to report more pronounced satisfaction [24], indicating that the benefits of ICCS may be more closely aligned with parental priorities or needs.

## Discussion

Understanding and learning from service users’ experiences is essential for developing effective mental health services that not only meet patients’ needs but are also desirable to them and promote treatment adherence. This review aimed to understand the experiences of stakeholders in regard to ICCS, and to gain insight into practical recommendations that could guide the development of similar services. Whilst our search did not find any articles on the experiences of staff working in ICCS, the reports by children and parents indicate general positive experiences with the support they received. ICCS models have generated sustained improvements through strong therapeutic alliance, a focus on positive interpersonal relationships, high clinical quality, and providing a variety of supports that cater to the needs of children and families. Our findings also highlighted potential pitfalls of excessive parental involvement that services need to carefully navigate.

### Importance of therapeutic alliance

For many young people, the quality of the therapeutic relationship with a clinician was the primary factor shaping their perceptions of mental health services, more so than any clinical outcomes. The quality of this therapeutic alliance was a prerequisite for any positive experiences. This is consistent with past research showing that therapeutic alliance is one of the strongest predictors of treatment outcomes and service user satisfaction in mental health care [28].It is important that clinicians present themselves as warm and authentic [29], as service users tend to value feeling heard and respected over specific interventions. The importance of having clinician who can build effective therapeutic relationship has been reported for other generic community mental health models [30, 31]. However, unlike traditional outpatient models where contact may be limited to weekly appointments, ICCS provide frequent, flexible contact in community and/or home settings. This may allow rapport to develop more naturally and organically. Clinicians entering the young person’s familiar environment rather than requiring them to come to a clinical setting helps establish a less hierarchical, more collaborative therapeutic relationship. It also models positive interpersonal skills and promotes a sense of social connectedness, which are important for young people whose mental illness may have temporarily removed them from education and peers.

Indeed, feeling less alone is a common theme in the feedback from not only young people but also their parents. Caregivers are the critical support system for children and adolescents and spend the most time with them, helping them with daily life and continuing treatment plans outside clinical sessions. Previous studies emphasised the protective effects of supportive family relationships against relapses of self-harm in young people [32]. However, parental well-being and, in turn, family functioning often suffer in the face of the child’s mental illness, creating a vicious cycle that hinders recovery. For greater treatment success, it is important to ensure that caregivers are also in good condition to provide support [33]. Traditional alternative models, apart from family therapy-focused services, may not necessarily have the needs of caregivers in mind [34]. On the other hand, ICCS models are often designed to be flexible, have families in mind, and provide wraparound support that helps problem-solve daily issues, all of which are proven effective engagement strategies [35]. Our review observed high levels of belief among parents in their child’s treatment, as well as willingness to participate and coordinate. This demonstrates that the strong therapeutic alliance extends to the family members around the child, building collaborative relationships that facilitate treatment.

### Careful calibration of family involvement

Despite the benefits of involving families in treatment, it is important to remember that young people want to be active participants in their care rather than passive recipients [36]. Our review found that family tension can be a potential challenge in the community, particularly when there is extensive parental involvement. Whilst a cohesive family dynamic is a protective factor, family discord, on the other hand, can undermine progress in the child’s recovery. Some children and adolescents reported family conflicts as an additional burden, whilst some described feeling like a bystander in their own treatment. Whilst these reports are fortunately in the minority, combined with the generally more pronounced parental satisfaction, our findings raise the question of whether services sometimes over-cater to parental needs at the expense of the adolescent sense of agency. If so, how can we balance between parental involvement and the young person’s autonomy? The answer perhaps lies within two contrasting experiences reported by some young people: feeling their underlying psychological distress is being overlooked, versus feeling reduced to their mental health problems. They are a reminder of young people’s wish to be truly heard and known as a person [37]. It is important to put in effort to co-develop the treatment with young people, instead of having a predetermined, fixed idea of what needs or does not need to be addressed. Sometimes this may be difficult at the early stage when a patient’s mental capacity is being affected by severe illnesses, especially considering the likely demographic of ICCS. Nevertheless, autonomy should be returned to young people gradually when they have the capacity, so that they can be empowered to advocate for their own needs and concerns. There also needs to be a focus on developing healthy family dynamics. This is where ecological family models may provide a helpful blueprint for ICCS models, as there is similarly an emphasis on providing family-centred care that calibrates to the child’s developmental stage and autonomy [38]. ICCS clinicians are well-placed to identify and resolve unhealthy patterns. Our review observed that, where treatment conflict management and communication skills were successfully acquired, there was an increase in sense of connectedness between children and their parents [20]. Children and adolescents reported that the treatment experience made them witness their parents not giving up on them, and realised they were on the same front, fighting the same battle. This demonstrates the potential that family tension can be transformed into opportunities for patients and parents to learn to collaborate to overcome in the community.

### Quality of clinical expertise

The availability of a variety of high-quality care is a strength of ICCS models. Being multi-disciplinary, these services are able to provide an individualised treatment plan combining relevant forms of expertise [6]. Children and adolescents can access multiple forms of support (e.g. psychiatric prescription, psychological therapy, behavioural support, social care liaison, etc.) without needing to navigate through different services and pathways. This minimises potential fatigue of retelling stories and familiarising with multiple clinicians and teams, which could reduce service engagement [39]. In contrast, in a generic community mental health model, there is commonly a disconnect and/or ineffective communication between different services, compromising treatment quality [40]. The provision of various treatment modalities also adds to the flexibility of ICCS and allows clinicians to integrate or switch to a different intervention when the one proves to be ineffective. Service users reported being very satisfied with the clinical expertise, with the bonus of treatment taking place in the community, displaying a level of coordination and intensity that matches the alternative inpatient setting. The quality of the service generates an inherent trust in children and adolescents towards the clinicians as well as the therapeutic exercises they introduce, therefore translating into higher treatment compliance both during and after the treatment.

### Sustained recovery beyond treatment

Our review found that parents and children tended to feel positive about continuing recovery after the support from ICCS ended. Children and adolescents reflected feeling confident to generalise calming strategies to other problems in life. Direct clinical contacts in the community helped scaffold parents’ ability to manage their child’s mental difficulties. Parents reported satisfaction with the techniques they learned and felt more equipped for life beyond treatment. These demonstrate the value of implementing rehabilitation in the community, as it provides a relevant setting for managing any future difficulties.

Successful mental health treatment not only targets clinical symptoms but also actively manage extra-therapeutic factors, such as family dynamics, influences from schools, peers, and the wider community, as well as one’s maturity level and physical health. These are particularly significant after support ends as they can persist or become new sources of stress. Reviews of adolescent inpatient care indicate that, while they are effective in stabilising acute symptoms, there is often a lack a clear focus on mechanisms of change or broader ecological recovery, including family and school contexts [41]. Young people often feel not given real opportunities and autonomy in psychiatric wards, in turn reporting a lack of real improvements that would generalise to the community [42]. The ecological approach of many ICCS models sets them apart from alternative community care or inpatient hospitalisation, which may only focus on certain symptoms whilst having limited insight into or capacity to address the young person’s broader life context. By working in homes, schools, and communities, ICCS clinicians can directly observe and intervene with the environmental factors that shape outcomes, preparing families to manage them beyond treatment and minimise relapse.

### Strengths and limitations

This is the first systematic review to address a gap in the literature regarding subjective experiences and satisfaction of ICCS models. Intensive alternatives to inpatient treatment for children and adolescents who have severe mental health difficulties are a rapidly growing area of interest both academically and clinically. Our meta-aggregative approach produced concrete generalisable statements that enabled us to identify important experiences that will guide future service development. This review adopted a comprehensive search strategy and a robust analytical approach. Both qualitative and quantitative findings were integrated in accordance with JBI mixed-methods review recommendations, enabling a deeper understanding of the existing evidence [43]. Four reviewers were involved in all stages, minimising researcher biases in screening, extraction, and analysis.

Several limitations need to be considered when interpreting our findings. First, although this review set out to include staff experiences of ICCS, we did not encounter any relevant published research. Clinicians are an essential element in any healthcare service. Their experiences delivering treatment as well as their perspectives on any associated benefits or challenges, in contrast to those of service users, would help identify systemic factors such as implementation barriers and resource requirements. Second, the included studies only measured global ratings of satisfaction rather than distinguishing between specific dimensions, limiting a nuanced understanding of service quality [44]. While we qualitised these quantitative satisfaction ratings and analysed them alongside qualitative data, this approach still cannot capture whether individuals were satisfied with certain aspects of care but dissatisfied with others. Third, we did not find any studies in any low- to middle-income countries. This might reflect the lack of equivalent services developed outside of the included few countries, or simply the lack of research on these services. Either way, it remains unclear whether our findings apply to services and populations in other cultural or economic settings. Fourth, the meta-aggregation approach comes with the limitation of being descriptive rather than interpretive. Our review could not be used to develop new theoretical frameworks beyond what is present in the original data. The theoretical model of ICCS remain unstudied and require further efforts to establish a clear operational framework. Future review may utilise a different approach, such as meta-ethnography, when there is richer qualitative information in the literature.

## Conclusion

This review identified many key benefits of ICCS models experienced by service users and provided insights into future effective clinical practice. Services should continue to prioritise building a strong therapeutic alliance with both children and parents. Training for staff can focus on relational skills as they are the foundation for any effective treatment. Although ICCS provide an advantageous setting for increased parent responsibilities in the child’s treatment, it needs to remain patient-centred. The perspectives of children and adolescents should not be neglected, and potential family tension should be carefully mediated. This can be achieved through collaborative goal setting that balances parental involvement and the autonomy of the young person. In severe cases where this is not possible at the start of treatment, clinicians should regularly assess and advocate for the child’s voice as soon as mental capacity is restored. In order to generate sustainable improvements, a comprehensive assessment is necessary to map out factors in a young person’s life that warrant further support or can be leveraged beyond treatment.

## Supplementary Information

Below is the link to the electronic supplementary material.

Supplementary Material 1

## Data Availability

Data extracted from included studies; data used for all analyses; analytic code; any other materials used in the review may be obtained from the corresponding author upon reasonable request. Template data collection forms can be found in the Supplementary materials.

## Declarations

### Ethnical information

This study is a systematic review and no ethical approvals were required, as all studies and information included in this study are published, publicly accessible data for which ethical approval had already been sought.

### Pre-registration

The current systematic review with meta-analysis was registered in the International Prospective Register of Systematic Reviews (PROSPERO) (CRD42024541495), register date 22 May 2024. Available from https://www.crd.york.ac.uk/PROSPERO/view/CRD42024541495.
